# Unravelling the truth: Examining the evidence for health-related claims made by naturopathic influencers on social media – a retrospective analysis

**DOI:** 10.34172/hpp.2022.49

**Published:** 2022-12-31

**Authors:** Van Nguyen, Luke Testa, Andrea L Smith, Louise A. Ellis, Adam G. Dunn, Jeffrey Braithwaite, Mitchell Sarkies

**Affiliations:** ^1^Australian Institute of Health Innovation, Macquarie University, Sydney, NSW, Australia; ^2^The Daffodil Centre, University of Sydney, Sydney, NSW, Australia; ^3^Biomedical Informatics and Digital Health, Faculty of Medicine and Health, The University of Sydney, Sydney, NSW, Australia

**Keywords:** Complementary therapies, Linear models, Naturopathy, Social media

## Abstract

**Background:** Social media platforms are frequently used by the general public to access health information, including information relating to complementary and alternative medicine (CAM). The aim of this study was to measure how often naturopathic influencers make evidence-informed recommendations on Instagram, and to examine associations between the level of evidence available or presented, and user engagement.

**Methods:** A retrospective observational study using quantitative content analysis on health-related claims made by naturopathic influencers with 30000 or more followers on Instagram was conducted. Linear regression was used to measure the association between health-related posts and the number of Likes, and Comments.

**Results:** A total of 494 health claims were extracted from eight Instagram accounts, of which 242 (49.0%) were supported by evidence and 34 (6.9%) included a link to evidence supporting the claim. Three naturopathic influencers did not provide any evidence to support the health claims they made on Instagram. Posts with links to evidence had fewer Likes (B=-1343.9, 95% CI=-2424.4 to -263.4, X=-0.1, *P*=0.02) and fewer Comments (B=-82.0, 95% CI=-145.9 to -18.2, X=-0.2, *P*=0.01), compared to posts without links to evidence. The most common areas of health were claims relating to ‘women’s health’ (n=94; 19.0%), and ‘hair, nail and skin’ (n=74; 15.0%).

**Conclusion:** This study is one of the first to look at the evidence available to support health-related claims by naturopathic influencers on Instagram. Our findings indicate that around half of Instagram posts from popular naturopathic influencers with health claims are supported by high-quality evidence.

## Introduction

 Health misinformation on social media is a serious public health concern. The rapid, unregulated dissemination of false health-claims can result in misinformed individuals who make decisions for themselves and their families that can have deleterious consequences.^1^ Inaccurate health information can cause harm by influencing the decisions people make about their health, and alternative therapies that replace evidence-based health can lead to adverse consequences.^2^

 Social media platforms have emerged as popular sources of health-related information for the general public.^3^ Here, people are exposed to self-management health advice, support and self-tracking of personal health and fitness with other community members.^4^ With few legal regulations governing the distribution of poor-quality information, social media provides a dynamic forum for circulating health misinformation.^5^

 One of the ways in which social media is being used is to discuss health information in relation to complementary and alternative medicine (CAM), including naturopathy.^6^ Although the training and regulation of naturopathic practitioners vary worldwide, naturopathic clinical education in many countries include treatment methods such as lifestyle-oriented self-care, dietary nutrition, homeopathy, herbal medicine, and over-the-counter medicines.^7^ Qualified clinicians, including naturopaths, should be providing evidence-informed advice to their patients and be careful in declaring conflicts of interest, and this should extend to online spaces.^8^ To date, little has been reported in the literature regarding health-related claims made on social media, especially on Instagram.^5^ Previous studies exploring health related information on Instagram specifically have focussed on HPV,^9^ vaping,^10^ COVID-19,^11^ and Zika virus.^12^ However, the validity of health-related claims made by naturopathic influencers on Instagram remains unknown. Previous research has highlighted concerns about low quality health information on social media and its impact on health outcomes.^13^ As the use of social media to seek health information grows, so do the concerns over the validity of online health information.^14^

 The aim of this study was to measure how often naturopathic influencers make evidence-informed recommendations on Instagram, and to examine associations between the level of evidence available or presented, and user engagement. The study objectives were to: (1) determine the proportion of health-related claims posted by naturopathic influencers that are supported by readily available high-quality evidence or include a link to sources of evidence; and (2) identify whether evidence-based health claims generate a higher or lower number of Instagram Likes and Comments relative to their number of Followers.

## Materials and Methods

###  Study design and cohort selection

 A retrospective observational study using quantitative content analysis on health-related claims made by naturopathic influencers on Instagram was conducted. Social media accounts are dynamic: new accounts are frequently created, and their number of followers changes continuously. An interactive sampling approach was therefore used to obtain a sample of Instagram accounts managed by naturopathic influencers. Initially, a seeding set of Instagram accounts from a public website that provides resources to consumers interested in naturopathic and alternative treatments was used as a starting point.^15^ From this list of Instagram accounts, the ‘suggested for you’ function on Instagram was used as a snowballing tool to identify additional contemporary Instagram accounts that met our inclusion criteria, as of January 2020. Instagram accounts were included in this study if they met the following criteria: (a) accounts held by individuals claiming to be a naturopathic doctor in the ‘Bio’ section of their Instagram account, and (b) accounts with at least 30 000 followers. We chose to only include accounts where qualifications were specified because the naturopathic influencers were more likely to be viewed as a trusted source of information by the public, and we expected these individuals to provide health advice that is supported by evidence. A cut-off of 30 000 followers was chosen. This is a level considered sufficient to constitute a celebrity endorsement for medicines by the UK Advertising Standards Authority.^16^

###  Data variable extraction and definition

 Health-related claims extracted from Instagram posts were converted to a clinical question using the PICO (population, intervention, comparison, outcome) framework to quantify the number of health-related claims present within each post.^17^ For example, where an Instagram post contained a claim that ‘beetroot improves liver function’, this was converted to P = general population, I = beetroot, C = placebo, O = improves liver function. The number of public Likes and Comments for each Instagram post containing a health-related claim was also recorded.

 The number of health claims formulated for each Instagram post was dependent on the number of interventions and outcomes mentioned in the post. For each health claim, we searched PubMed using Medical Subject Headings (MeSH) and non-MeSH terms. The search results were filtered to show only human studies and studies published before the Instagram post. It was beyond the scope of this study to conduct a systematic review for each health claim, so the first 10 articles ranked by relevance on PubMed were retrieved and reviewed for whether they produced a conclusion that aligned with the claim to serve as a proxy for relevant literature. Those articles were then assigned a level of evidence using the National Health and Medical Research Council (NHMRC) levels of evidence for intervention studies (Supplementary file 1).^18^ Studies were considered to refute the claim where reported findings contradicted the health claim made on the Instagram post.

 Data collection commenced in January 2020, covering a six-month retrospective period to account for potential seasonal effects in the topics of Instagram posts. To be included in this study, Instagram posts must have been dated between 1 June 2019 and 31 December 2019, with posts containing a health claim promoting a health intervention. Video posts and those that related to a risk-based exposure, rather than an intervention, were excluded (e.g., ‘bisphenol A (BPA) is associated with lower fertility and increased risk of miscarriage’). To reduce the risk of temporal bias in relation to the number of Followers, Likes and Comments captured during data collection, data extraction for each naturopathic influencer was performed for the same month before moving on to the subsequent month.

 The data variables of interest were:

1. The highest level of evidence to support or refute health-related claims posted on Instagram by naturopathic influencers, graded using the NHMRC levels of evidence for intervention studies.^18^
2. Number of health-related posts that contained a link to sources of evidence. A link to source(s) of evidence is provided if the naturopathic influencer mentioned the name of the study, author(s) of the study or provided a direct web link to the published study.
3. Level of evidentiary support: sources of evidence provided or identified in PubMed were evaluated using the NHMRC levels of evidence.^18^
4. The number of public Likes and Comments for each Instagram post containing a health-related claim.

###  Statistical analysis

**Figure 1:**
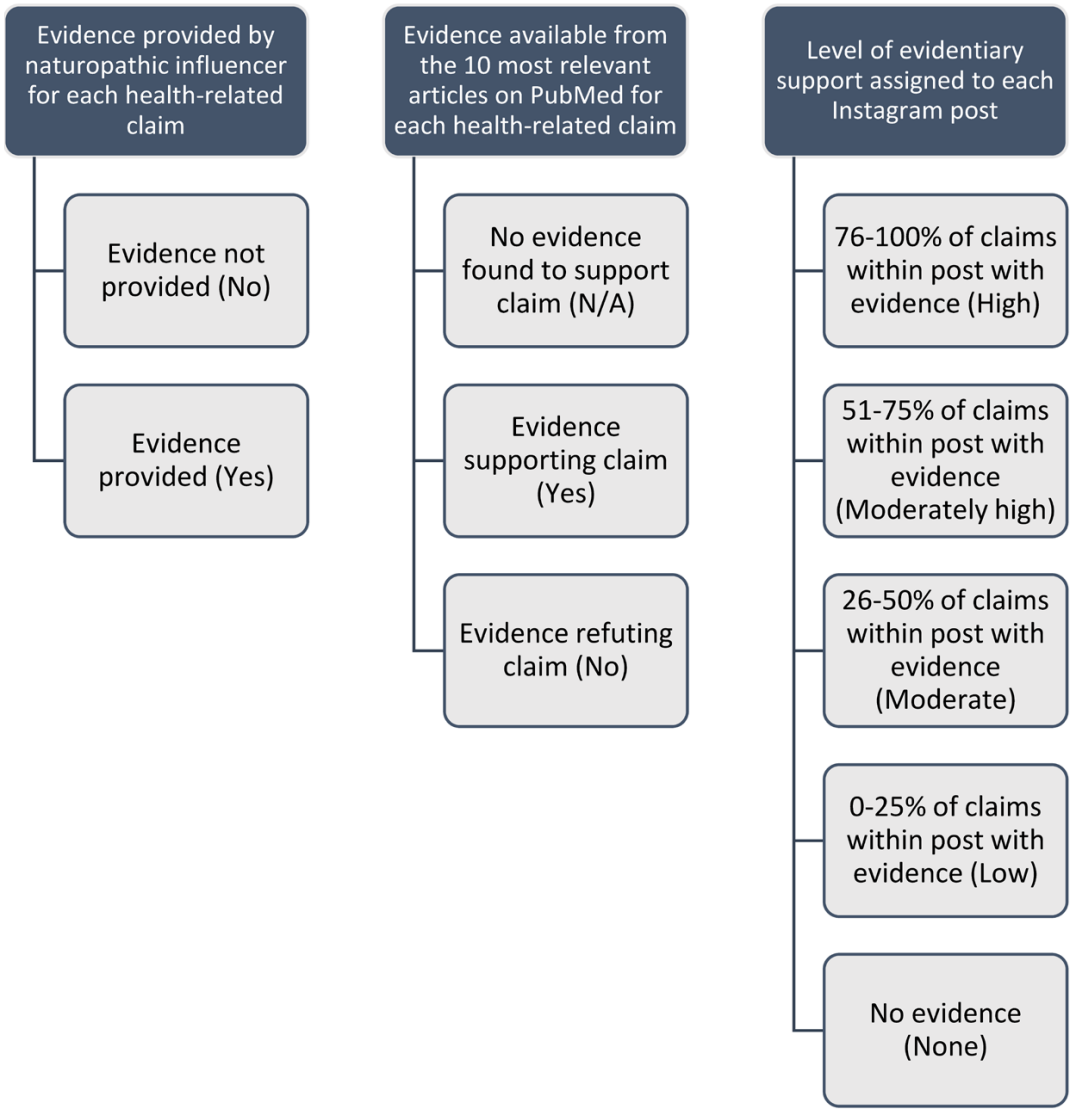
Levels of evidence assigned for each health-related claim and Instagram post

 Descriptive summative statistics were used to present whether evidence was provided by naturopathic influencers (binary: no; yes) and the evidence available from the 10 most relevant articles on PubMed for health claims posted by each individual naturopathic influencer (three-level: no evidence identified; supporting evidence identified; refuting evidence identified) and for different health claim categories (Figure 1). To analyse the relationship between health-related Instagram posts and the number of Likes and Comments, the level of evidentiary support for posts, for which evidence was found, were divided into five categories: high, moderately high, moderate, low, and none. Level of evidentiary support for post was assigned, as follows: high = 76-100% of claims within post with evidence; moderately high = 51-75% of claims within post with evidence; moderate = 26-50% of claims within post with evidence; low = 0-25% of claims within post with evidence, none = no evidence.

 Linear regression was computed to measure the association between health-related posts and the number of Likes, and Comments. No evidence and refuting evidence were collapsed into one category as ‘no evidentiary support’ due to the small number of claims identified with refuting evidence. The Likes and Comments were entered into the model as dependent variables while evidentiary support for claim (no; yes), highest evidence level identified and level of evidence provided by naturopathic influencer (high, moderately high, moderate, low, and none, based on NHMRC levels of evidence), link to evidence provided by naturopathic influencer (no; yes), and health claim category were entered as independent variables, number of Followers was entered as a control variable. Collinearity was assessed to ensure the value of tolerance was less than 1.0 for each analysis. Residual plots were also performed to ensure a linear relationship between the logit of the outcome and each predictor variable. Statistical significance was set at α = 0.05. Statistical analysis was performed using IBM SPSS Statistics V.25.0.^19^

## Results

 A total of nine Instagram accounts met the inclusion criteria of ≥ 30 000 followers. Of these nine accounts, one was excluded from data extraction due to posting only video content. From the remaining eight accounts, a total of 494 health claims were extracted for data analysis. Over half (51.0%) of the 494 health claims had no evidence available (Table 1). Regarding the claims with evidence available, 10.9% (n = 54/494) were systematic reviews of randomised controlled trials (Level I), 19.8% (n = 98/494) were randomised controlled trials (Level II), 0.2% (n = 1/494) were pseudorandomised controlled trials (Level III-1), 10.5% (n = 52/494) were comparative studies with concurrent controls (Level III-2), 5.5% (n = 27/494) were comparative studies without concurrent controls (Level III-3) and 2.0% (n = 10/494) were based on case studies (Level IV).

**Table 1:** Highest level of evidence found on PubMed for health claims posted by naturopathic influencers on Instagram

**Naturopath**	**NHMRC level of evidence **							
	**I**	**II**	**III-1**	**III-2**	**III-3**	**IV**	**Nil**	**Total **
	**No. (%)**	**No. (%)**	**No. (%)**	**No. (%)**	**No. (%)**	**No. (%)**	**No. (%)**	**No. (%)**
1	2 (4.1)	5 (10.2)	1 (2.0)	7 (14.3)	1 (2.0)	1(2.0)	32 (65.3)	49 (100)
2	16 (13.7)	23 (19.7)	0 (0.0)	16 (13.7)	8 (6.8)	3 (2.6)	51 (43.6)	117 (100)
3	9 (16.7)	6 (11.1)	0 (0.0)	6 (11.1)	1 (1.9)	2 (3.7)	30 (55.6)	54 (100)
4	4 (9.8)	6 (14.6)	0 (0.0)	7 (17.1)	4 (9.8)	2 (4.9)	18 (43.9)	41 (100)
5	5 (8.6)	13 (22.4)	0 (0.0)	3 (5.2)	2 (3.4)	2 (3.4)	33 (56.9)	58 (100)
6	6 (22.2)	12 (44.4)	0 (0.0)	3 (11.1)	2 (7.4)	0 (0.0)	4 (14.8)	27 (100)
7	1 (4.3)	7 (30.4)	0 (0.0)	2 (8.7)	1 (4.3)	0 (0.0)	12 (52.2)	23 (100)
8	11 (8.8)	26 (20.8)	0 (0.0)	8 (6.4)	8 (6.4)	0 (0.0)	72 (57.6)	125 (100)
Total	54 (10.9)	98 (19.8)	1 (0.2)	52 (10.5)	27 (5.5)	10 (2.0)	252 (51.0)	494 (100)

NHMRC: National Health and Medical Research Council; Naturopath: Naturopathic influencer. Note: Levels of evidence were graded using the NHMRC levels of evidence for intervention studies, which is graded from: I = a systematic review of level II studies; II = a randomised controlled trial; III-1 a pseudorandomised controlled trial; III-2 = a comparative study with concurrent controls; III-3 = a comparative study without concurrent controls; IV = case series with either post-test or pre-test/post-test outcomes.

 From the total number of health claims extracted, 6.9% (n = 34/494) of health claims provided Instagram users with a link to evidence to support the claim made (Table 2). Three out of the eight naturopathic influencers provided no evidence to support any of the health claims they made on Instagram. When evidence was provided to support the claims made, it was most commonly in the form of a randomised controlled trial (Table 3).

**Table 2:** Link to evidence supporting health claim provided by naturopathic influencer

**Naturopath**	**No**	**Yes**	**Total**
	**No. (%)**	**No. (%)**	**No. (%)**
1	49 (100.0)	0 (0.0)	49 (100)
2	117 (100.0)	0 (0.0)	117 (100)
3	37 (68.5)	17 (31.5)	54 (100)
4	36 (87.8)	5 (12.2)	41 (100)
5	57 (98.3)	1 (1.7)	58 (100)
6	18 (98.3)	9 (33.3)	27 (100)
7	21 (91.3)	2 (8.7)	23 (100)
8	125 (100.0)	0 (0.0)	125 (100)
Total	460 (93.1)	34 (6.9)	494 (100)

Naturopath: Naturopathic influencer.

**Table 3:** Quality of evidence provided by naturopathic influencer to support health claims posted on Instagram

**Naturopath**	**NHMRC level of evidence**						
	**I**	**II**	**III-1**	**III-2**	**III-3**	**IV**	**N/A**
	**No. (%)**	**No. (%)**	**No. (%)**	**No. (%)**	**No. (%)**	**No. (%)**	**No. (%)**
1 (n = 0)	0 (0.0)	0 (0.0)	0 (0.0)	0 (0.0)	0 (0.0)	0 (0.0)	0 (0.0)
2 (n = 0)	0 (0.0)	0 (0.0)	0 (0.0)	0 (0.0)	0 (0.0)	0 (0.0)	0 (0.0)
3 (n = 17)	0 (0.0)	3 (17.6)	0 (0.0)	4 (23.5)	1 (5.9)	2 (11.8)	7 (41.2)
4 (n = 5)	0 (0.0)	5 (100.0)	0 (0.0)	0 (0.0)	0 (0.0)	0 (0.0)	0 (0.0)
5 (n = 1)	0 (0.0)	0 (0.0)	0 (0.0)	1 (100.0)	0 (0.0)	0 (0.0)	0 (0.0)
6 (n = 9)	5 (55.6)	1 (11.1)	0 (0.0)	1 (11.1)	1 (11.1)	1 (11.1)	0 (0.0)
7 (n = 2)	0 (0.0)	2 (100.0)	0 (0.0)	0 (0.0)	0 (0.0)	0 (0.0)	0 (0.0)
8 (n = 0)	0 (0.0)	0 (0.0)	0 (0.0)	0 (0.0)	0 (0.0)	0 (0.0)	0 (0.0)
Total (n = 34)	5 (14.7)	11 (32.4)	0 (0.0)	6 (17.6)	2 (5.9)	3 (8.8)	7 (20.6)

NHMRC: National Health and Medical Research Council; Naturopath: Naturopathic influencer; N/A: not applicable (evidence involving pre-clinical studies.)

 Claims related to ‘women’s health’ (19.0%, n = 94/494) and ‘hair, nail and skin’ (15.0%, n = 74/494) were the two most identified health claim categories (Table 4). However, more than half of the claims had no evidence for either of these health claim categories. Claims related to mental health had the highest proportion of posts with NHMRC Level I (n = 13/50, 26%) and Level II evidence (n = 14/50, 28%). Claims related to ‘oral health’ (0% with evidence, n = 0/1), ‘medications’ (0% with evidence, n = 0/5) and ‘eye health’ (6.2% with evidence, n = 1/16) had the lowest proportion of claims with evidence available.

**Table 4:** Highest level of evidence found on PubMed for health claims based on health claim category

**Health claim category**	**NHMRC Level of Evidence**						
	**Nil**	**I**	**II**	**III-1**	**III-2**	**III-3**	**IV**
	**No. (%)**	**No. (%)**	**No. (%)**	**No. (%)**	**No. (%)**	**No. (%)**	**No. (%)**
Brain health (n = 20)	10 (50.0)	4 (20.0)	2 (10.0)	0 (0.0)	3 (15.0)	1 (5.0)	0 (0.0)
Cancer (n = 6)	2 (33.3)	2 (33.3)	1 (16.7)	0 (0.0)	1 (16.7)	0 (0.0)	0 (0.0)
Children’s health (n = 1)	0 (0.0)	0 (0.0)	0 (0.0)	0 (0.0)	0 (0.0)	1 (100.0)	0 (0.0)
Cold, flu & immunity (n = 24)	12 (50.0)	5 (20.8)	4 (16.7)	0 (0.0)	1 (4.2)	2 (8.3)	0 (0.0)
Digestive health (n = 32)	18 (56.3)	3 (9.4)	8 (25.0)	0 (0.0)	2 (6.3)	0 (0.0)	1 (3.1)
Endocrine health (n = 15)	6 (40.0)	2 (13.3)	5 (33.3)	0 (0.0)	2 (13.3)	0 (0.0)	0 (0.0)
Energy & exercise (n = 16)	7 (43.8)	2 (12.5)	3 (18.8)	0 (0.0)	2 (12.5)	1 (6.3)	1 (6.3)
Eye health (n = 16)	15 (93.8)	0 (0.0)	1 (6.3)	0 (0.0)	0 (0.0)	0 (0.0)	0 (0.0)
General health (n = 45)	22 (48.9)	4 (8.9)	13 (28.9)	0 (0.0)	5 (11.1)	1 (2.2)	0 (0.0)
Hair, nail & skin (n = 74)	43 (58.1)	3 (4.1)	12 (16.2)	0 (0.0)	5 (6.8)	9 (12.2)	2 (2.7)
Heart & circulation (n = 14)	5 (35.7)	3 (21.4)	5 (35.7)	0 (0.0)	1 (7.1)	0 (0.0)	0 (0.0)
Joint, bone & muscle (n = 23)	11 (47.8)	2 (8.7)	6 (26.1)	0 (0.0)	4 (17.4)	0 (0.0)	0 (0.0)
Medications (n = 5)	5 (100.0)	0 (0.0)	0 (0.0)	0 (0.0)	0 (0.0)	0 (0.0)	0 (0.0)
Men’s health (n = 4)	2 (50.0)	0 (0.0)	0 (0.0)	0 (0.0)	2 (50.0)	0 (0.0)	0 (0.0)
Mental health (n = 50)	16 (32.0)	13 (26.0)	14 (28.0)	0 (0.0)	4 (8.0)	2 (4.0)	1 (2.0)
Oral health (n = 1)	1 (100.0)	0 (0.0)	0 (0.0)	0 (0.0)	0 (0.0)	0 (0.0)	0 (0.0)
Pregnancy & pre-conception (n = 34)	14 (41.2)	2 (5.9)	6 (17.6)	0 (0.0)	7 (20.6)	4 (11.8)	1 (2.9)
Sleep (n = 16)	8 (50.0)	1 (6.3)	4 (25.0)	0 (0.0)	2 (12.5)	1 (6.3)	0 (0.0)
Weight loss (n = 4)	1 (25.0)	0 (0.0)	2 (50.0)	1 (25.0)	0 (0.0)	0 (0.0)	0 (0.0)
Women’s health (n = 94)	54 (57.4)	8 (8.5)	12 (12.8)	0 (0.0)	11 (11.7)	5 (5.3)	4 (4.3)

**Figure 2:**
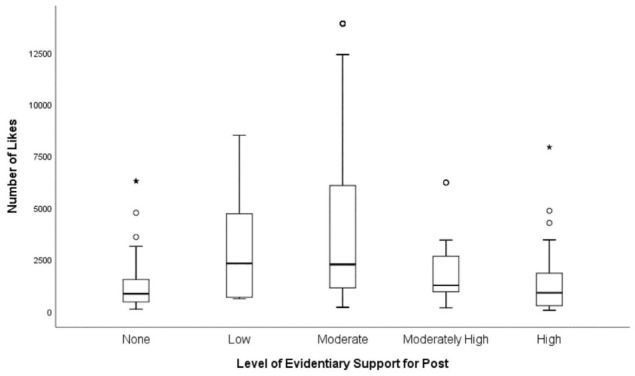
Box plot showing level of evidentiary support for Instagram post and the number of likes.Level of evidentiary support for post: high = 76-100% of claims within post with evidence; moderately high = 51-75% of claims within post with evidence; moderate = 26-50% of claims within post with evidence; low = 0-25% of claims within post with evidence, none = no evidence. Circles and asterisks represent outliers

**Figure 3:**
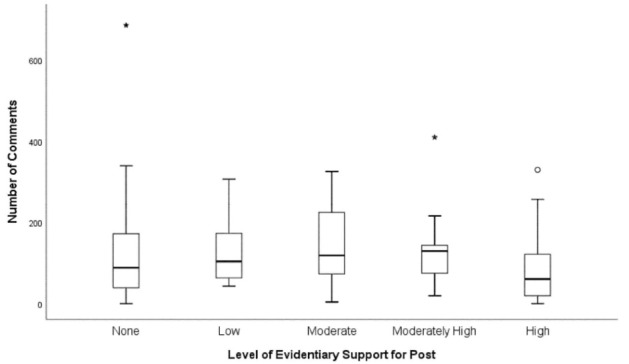
Box plot showing level of evidentiary support for Instagram post and the number of comments. Level of evidentiary support for post: high = 76-100% of claims within post with evidence; moderately high = 51-75% of claims within post with evidence; moderate = 26-50% of claims within post with evidence; low = 0-25% of claims within post with evidence, none = no evidence. Circles and asterisks represent outliers

 Posts with low and moderate levels of evidentiary support had the highest median number of Likes (Figure 2). Posts with a moderate level of evidentiary support had the greatest distribution in the number of Likes. A condensed spread of Likes is seen for post with no evidence (Figure 2). Posts with a high level of evidentiary support had the lowest median number of Comments (Figure 3). For posts with none, low, moderate, and moderately high level of evidentiary support, the median number of Comments increased with increasing levels of evidentiary support (Figure 3).

 After controlling for the number of followers, posts with links to evidence had fewer Likes (β = -0.1, *P* = 0.02) and fewer Comments (β = -0.2, *P* = 0.01), compared to posts without links to evidence (Table 5). Health claims categorised under ‘general health’ with evidentiary support were associated with a higher number of Likes (β = -0.4, *P* < 0.001) and Comments (β = 0.3, *P* = 0.03), which included issues related to anti-aging, increasing in-vivo collagen production and reducing in vivo inflammatory responses. Health claims with evidentiary support for topics related to ‘cold, flu and immunity’ were associated with approximately 225 more Comments than claims within the same category without evidentiary support (β = 0.4, *P* < 0.001).

**Table 5:** Relationship between number of Instagram likes and comments, and evidentiary support for health claims, link to evidence and health claim category

	**Instagram Likes**				**Instagram Comments**			
	**B**	**Std error**	**β**	**95% CI**	**B**	**Std error**	**β**	**95% CI**
Evidentiary support for claim	-148.9	215.4	0.0	-572.3–274.5	-12.4	12.7	-0.1	-37.5–12.6
Highest evidence level identified	68.9	85.9	0.0	-99.9–237.8	2.2	5.1	0.0	-7.8–12.2
Link to evidence provided	-1343.9	549.8	-0.1*	-2424.4 – -263.4	-82.0	32.5	-0.2*	-145.9 – -18.2
Level of evidence provided	492.7	256.4	0.1	-11.2–996.7	16.8	15.2	0.1	-13.0–46.6
Health claim category								
Brain health	-767.7	895.9	0.0	-2528.4–993.0	-15.8	52.9	0.0	-119.9–88.2
Cold, flu & immunity	1628.4	924.9	0.1	-189.2–3446.0	224.5	54.7	0.4**	117.0–331.9
Digestive health	800.3	880.7	0.1	-930.4–2531.1	32.3	52.0	0.1	-69.9–134.6
Endocrine health	74.2	961.3	0.0	-1815.1–1963.5	76.1	56.8	0.1	-35.5–187.8
Energy & exercise	245.4	957.5	0.0	-1636.2–2127.1	30.6	56.6	0.0	-80.6–141.8
Eye health	-523.7	900.4	0.0	-2293.3–1245.9	22.9	53.2	0.0	-81.7–127.5
General health	4476.8	891.5	0.4**	2724.6–6228.9	117.1	52.7	0.3*	13.5–220.6
Hair, nail & skin	-325.9	867.8	0.0	-2031.4–1379.7	15.6	51.3	0.0	-85.2–116.4
Heart & circulation	-1034.7	969.7	-0.1	-2940.4–870.9	-10.7	57.3	0.0	-123.3–101.9
Joint, bone & muscle	-919.2	921.4	-0.1	-2730.0–891.6	-35.9	54.4	-0.1	-143.0–71.1
Mental health	-3.2	883.8	0.0	-1740.1–1733.8	47.9	52.2	0.1	-54.8–150.5
Pregnancy & pre-conception	-61.9	902.1	0.0	-1834.8–1711.0	10.2	53.3	0.0	-94.6–115.0
Sleep	1109.3	952.0	0.1	-761.6–2980.3	49.9	56.3	0.1	-60.7–160.4
Women’s Health	238.5	873.5	0.0	-1478.1–1955.1	51.2	51.6	0.2	-50.3–152.6

* *P* < 0.05; ^**^P < 0.001.

## Discussion

 We found approximately half of the health claims posted on Instagram by naturopathic influencers with 30 000 or more followers were unsupported by evidence. Of those with evidence clearly available, approximately 10% of health claims were underpinned by high quality evidence such as systematic reviews of randomised controlled trials. Health claims were rarely accompanied by a link to supporting evidence. Posts that had evidence available received fewer Likes and Comments, whether the evidence was linked or if it was identified via a literature search.

 Little attention has been directed towards the credibility of health information available on social media despite evidence showing that social media use can influence health behaviours, decision-making, and risk perceptions.^20^ Many health claims on social media promote health remedies that are untested, ineffective, or unsafe, which may introduce unnecessary risks of harm.^21^ The results may be partially explained by the relatively small number of clinical trials undertaken for CAM, which are complicated by barriers such as lack of access to funding and lack of accessibility to well-trained scientists to conduct high-quality clinical trials.^22^ Although social media guidance exists for registered health practitioners in Australia, for example^23^; engagement in controversial topics on social media has been linked to an increase in public interest and discussion, generating higher amounts of traffic to the social media profile.^24^

 Lower engagement with claims supported by evidence or provided a link to supporting evidence signals a potential disinterest in evidence-based medicine by those Instagram users following naturopathic influencers. This may be explained by limited knowledge and understanding of the scientific process, issues with health literacy and digital health literacy,^25^ or may reflect a lack of trust or disengagement with established institutions and those with recognised expertise.^26^ Disinterest towards evidence-based medicine also is thought to be driven by a preference for lived experience over evidence from published research outlined in academic journal articles.^27^ To encourage the effective communication of evidence-based health information, the use of storytelling can improve engagement with evidence-based information, while increasing the chances for this information to be absorbed and retained.^28^

 Studies on other social media platforms have produced results that converge with our findings. A study investigating health information related to sun protection and skin cancer prevention on YouTube concluded that videos on tanning beds and sunscreen contained inaccurate information 40% and 20% of the time, respectively.^29^ A study exploring the dissemination of public health information on the Zika virus via Facebook found that misleading posts were far more popular than posts sharing accurate, relevant information.^30^ Additionally, other healthcare professionals, including physicians and dieticians, have also shared a considerable number of medical information on Twitter that were later concluded to be false based on expert review.^8^ In contrast to the health-related claims topics posted by naturopathic influencers in our study, a systematic review investigating the spread of health-related misinformation on social media generally found misinformation largely related to vaccines and communicable diseases, and to a lesser extent, chronic non-communicable diseases, such as cancer.^27^

 A key limitation was relying on the 10 most relevant retrieved articles in PubMed, rather than conducting an extensive search for evidence, when not provided alongside the claim. It is possible we did not identify existing evidence to support some of the claims; however, it would not have been feasible to construct a comprehensive search strategy and screen articles for every health claim made. Our search strategy served as a proxy measure for the evidence to support the claim and it is unlikely that the 10 most relevant articles on PubMed would systematically fail to identify existing supporting evidence.

 The risk of subjectivity in the extraction of interventional health claims from Instagram posts and conversion into a PICO is another limitation. The subjectivity of data extraction was minimised using standards which included extracting specific terms used by the naturopathic influencer to perform database searches on PubMed. If not explicitly stated, the intended target audience of the health claims were made using educated guesses based on the intervention and outcome promoted in the claim. For the claim to be considered supported by evidence, study participants must match the intended audience of the health claim on Instagram(e.g., postmenopausal women, athletes). When the study with the highest level of evidence were inconclusive due to reasons such as inadequate cohort size, conflicting results for different cohort (e.g., male vs. females), heterogeneity between studies and poor quality of studies, it was concluded that the evidence did not support the health claim. When there was considerable uncertainty related to the health intervention or outcome, it was excluded from the study.

 Our study captured a sample of naturopathic influencers, using an iterative approach. The population of potentially eligible Instagram accounts could not be known or specified at each stage of the sampling process. Accounts were only considered if they met our inclusion criteria. Verification and replication of the study sample is unlikely to be feasible given the dynamic nature of Instagram accounts. Therefore, repetition of this process may result in a slightly different sample. However, we do not believe this limits the representativeness and external validity of the sample identified.

 Claims were only extracted if they related to health interventions, and we excluded any claims related to a health risk exposure. Other limitations related to the sample size; including video posts or expanding the scope of influencers that could be included in the study may have revealed additional or alternative findings related to evidence or non-evidence-based posts on Instagram. It is not possible to determine the nature of the relationship between Instagram accounts providing evidence-based health claims and number of Instagram Likes and Comments from this study. The relationship may be due to the lack of interest in evidence-based information, the number of Followers the account already has, Followers viewing the claim as a form of advertisement, or other unknown reasons.

###  Clinical implications and future recommendations 

 The lack of evidence to support online health claims made on social media platforms such as Instagram is an important and understudied issue. Social media platforms allow inappropriate content to be reported, and this may be a useful way for social media platforms to partner with trusted authorities to identify and flag or downrank health claims that are unsubstantiated and may cause harm. Due to the highly variable quality of online health information, consumers may not be well-equipped with the skills to discern the quality of health information. There is potential for the use of automatic credibility appraisal tools to identify communities at higher risk of exposure to low-credibility health information on the internet.^31^ Given the influence of social media in healthcare, clinicians and researchers should focus on identifying the most effective ways of disseminating evidence-based health information on social media, populations most at risk of health misinformation and interventions to reduce the spread of health misinformation on social media.

## Conclusion

 This study is one of the first to look at the evidence available to support health-related claims on Instagram. Our findings highlight that around half of posts from popular naturopathic influencers with health claims that are supported by high-quality evidence. The dissemination and amplification of health misinformation, especially those that are counter to the policies and activities of public health organisations, can lead to harmful behaviours.

## Author Contributions

**Conceptualisation:** Andrea L Smith, Adam G. Dunn, Mitchell Sarkies.

**Methodology:** Van Nguyen, Andrea L Smith, Louise A. Ellis, Adam G. Dunn, Mitchell Sarkies.

**Investigation: **Van Nguyen.

**Formal analysis: **Van Nguyen, Luke Testa, Louise A. Ellis.

**Visualization: **Van Nguyen, Luke Testa.

**Writing–original draft preparation: **Van Nguyen.

**Writing–Review & Editing**: Luke Testa, Andrea L Smith, Louise A. Ellis, Adam G. Dunn, Jeffrey Braithwaite, Mitchell Sarkies.

**Supervision:** Andrea L Smith, Mitchell Sarkies.

## Funding

 No funding declared.

## Ethical Approval

 Ethical review was not required for this study as it did not involve human subjects.

## Competing Interests

 The authors have declared that there are no financial or non-financial competing interests

## Supplementary Files


Supplementary file 1. NHMRC levels of evidence for intervention studies.
