# Supplementary material for: Unravelling the truth: Examining the evidence for health-related claims made by naturopathic influencers on social media – a retrospective analysis
Source: Health Promot Perspect. 2022 Dec 31;12(4):372–80. doi: 10.34172/hpp.2022.49 (PMC9958238; doi:10.34172/hpp.2022.49)
Supplement: Supplementary file 1 — NHMRC levels of evidence for intervention studies. [file hpp-12-372-s001.pdf]

Nguyen et al, **Health Promotion Perspectives,** 2022, 12(4), 372-380
doi: 10.34172/hpp.2022.49
https://hpp.tbzmed.ac.ir

Unravelling the truth: Examining the evidence for health-related claims made by naturopathic influencers on social media – a retrospective analysis

Van Nguyen[1#], Luke Testa[1*#], Andrea L Smith[2], Louise A. Ellis[1], Adam G. Dunn[3], Jeffrey Braithwaite[1], Mitchell Sarkies[1]

[1]Australian Institute of Health Innovation, Macquarie University, Sydney, NSW, Australia
[2]The Daffodil Centre, University of Sydney, Sydney, NSW, Australia
[3]Biomedical Informatics and Digital Health, Faculty of Medicine and Health, The University of Sydney, Sydney, NSW, Australia

Supplementary File 1. NHMRC Levels of Evidence for Intervention Studies

Level of Evidence	Type of Study Design
I	A systematic review of Level II studies
II	A randomised controlled trial
III-I	A pseudorandomised controlled trial (i.e., alternate allocation of some other method)
III-2	A comparative study with concurrent controls:<br>• Non-randomised experimental trial<br>• Cohort study<br>• Case-control study<br>• Interrupted time series with a control group
III-3	A comparative study without concurrent controls:<br>• Historical control study

	• Two or more single arm study<br>• Interrupted time series without a parallel control group
IV	Case studies with either post-test or pre-test/post-test outcomes
